# Severe acute respiratory coronavirus virus 2 (SARS-CoV-2) IgG results among healthcare workers in a rural upstate New York hospital system

**DOI:** 10.1017/ice.2020.1296

**Published:** 2020-10-26

**Authors:** Wendy M. Brunner, Liane Hirabayashi, Nicole L. Krupa, Melissa B. Scribani, Paul Jenkins, Stephen D. Clark, John J. May, Anne M. Gadomski

**Affiliations:** 1 Research Institute, Bassett Medical Center, Cooperstown, New York; 2 Northeast Center for Occupational Health and Safety, Bassett Medical Center, Cooperstown, New York

*To the Editor*—To better understand the effectiveness of occupational infection control measures during the coronavirus disease 2019 (COVID-19) pandemic, we surveyed and antibody tested employees of Bassett Healthcare Network, located in rural upstate New York, 200 miles northwest of New York City. Employer-sponsored SARS-CoV-2 IgG antibody testing was conducted from May 4 to 29, 2020. Network leadership prioritized employees with a high likelihood of exposure to COVID-19–infected patients. A 21.7% random sample of employees was also included for antibody testing to ensure that our seroprevalence estimate was representative of all network employees.

The study questionnaire captured demographics, COVID-19 polymerase chain reaction (PCR) status, potential COVID-19 work and nonwork exposures, and COVID-19 symptoms experienced since January 1, 2020 (based on the Centers for Disease Control and Prevention’s case report form). The recall period for exposure questions (March 1–May 31, 2020) coincided with New York State’s stay-at-home order plus 2 weeks.

We compared seroprevalence among employees to that for patients tested during the same period. Employee serology testing was performed by Bassett Medical Center Laboratory using the SARS-CoV-2 IgG Abbott Architect assay (Abbott Laboratories, Abbott Park, IL), with 100% sensitivity and 99.6% specificity.^1^ IgG level ≥1.40 was defined as positive. Most serology testing for patients (96%) was conducted by the Mayo Clinic using the VITROS Anti-SARS-CoV-2 IgG Test (Ortho-Clinical Diagnostics, Linden, NJ), with 90.0% sensitivity and 100% specificity.^2^ The remaining patient tests were conducted by the in-house laboratory using the Abbott test.

We compared questionnaire responses by antibody status using χ^2^ tests for categorical variables and *t* tests for continuous variables. Analyses were conducted using SAS version 9.3 software (SAS Institute, Cary, NC). This study was approved by the Mary Imogene Bassett Institutional Review Board.

## Results

Among 764 employees tested, 15 were positive for SARS-CoV-2 IgG antibodies, for a seroprevalence of 2.0%, compared to 4.5% for patients (ie, 34 seropositives among 762 tested). Of the 764 employees tested, 601 (78.7%) completed the study questionnaire.

Seroprevalence among all study participants was 12 of 601 (2.0%) and 4 of 130 (3.1%) in the random sample. The mean IgG level was 4.93 (range, 2.10–8.14) among seropositives and 0.06 (range, 0.01–1.17) among seronegatives. One-third of seropositives reported a positive PCR test, 8.3% reported a negative PCR test, and 58.3% had no PCR test. Of the 589 seronegative employees, 47 reported having a PCR test (all negative).

Seropositive employees were just as likely as seronegative employees to report having no direct contact with COVID-19 patients (25.0% vs 17.7%; *P* = 0.46) (Table 1). Seropositive employees were less likely to report involvement in high-risk patient-related tasks, such as COVID-19 testing, although these differences were not statistically significant.

**Table 1.** Comparison of Employees With and Without Previous SARS-CoV-2 Infection (N=601), Bassett Healthcare Network, May 4–29, 2020

Characteristic	No. (%)	
	Positive IgG Test (N=12)	Negative IgG Test (N=589)
**Age**		
Mean y (standard deviation)	41.8 (16.0)	43.7 (13.7)
Range	23–63	19–78
**Sex**		
Female	10 (83.3)	425 (72.4)
Male	2 (16.7)	161 (27.6)
**Race/ethnicity**		
Black	1 (8.3)	12 (2.0)
White	10 (83.3)	521 (88.5)
Other	1 (8.3)	44 (7.4)
Body mass index, mean (standard deviation)	30.6 (11.1)	29.8 (7.3)
**Employment** ^a^		
Full-time	8 (66.7)	512 (88.4)
Part-time/Per diem	4 (33.3)	67 (11.6)
**Quarantined, yes^b^**	7 (58.3)	67 (11.4)
Diagnosed COVID positive	4 (33.3)	0
Exposed to known case	3 (25.0)	47 (8.0)
Travel	0	7 (1.2)
**Patient-related tasks**		
No direct patient contact	3 (25.0)	104 (17.7)
Testing for COVID-19 in a testing site	0	86 (14.6)
Testing for COVID-19 of patients being admitted	0	89 (15.1)
Emergency services	1 (8.3)	131 (22.2)
Bedside care of nonintubated patient	6 (50.0)	302 (51.3)
Intubation or extubation of patient	1 (8.3)	96 (16.3)
Intensive care of intubated patient	3 (25.0)	138 (23.4)
Respiratory therapy	0	42 (7.1)
Patient transport	1 (8.3)	94 (16.0)
Inpatient support—radiology, phlebotomy/IV team, physical/occupational/speech therapy	0	77 (13.1)
Servicing rooms	0	23 (3.9)
Food and nutrition services	0	8 (1.4)
Clinic rooming and patient visits	1 (8.3)	56 (9.5)
Clinic reception/ward clerk/clinic manager	0	26 (4.4)
Other patient care-related	1 (8.3)	62 (10.5)
Temperature monitoring of employees and patients at entrances	0	38 (6.5)
Respirator fit testing	0	19 (3.2)
**Possible nonwork exposures**		
Traveled out of region	2 (16.7)	93 (15.9)
Social distancing		
Strict/some	11 (91.7)	551 (96.3)
Not very much/none	1 (8.3)	21 (3.7)
Any nonwork contact with a known/suspected COVID-19 patient^c^	8 (66.7)	213 (37.6)
Had any contact (within 2 m for >15 min) with a known/suspected COVID-19 patient	5 (41.7)	181 (31.6)
Known	4 (80.0)	108 (60.0)
Suspected	1 (20.0)	62 (34.3)
Lived with a known/suspected COVID-19 patient^d^	5 (41.7)	62 (10.9)
Known	3 (60.0)	11 (17.7)
Suspected	1 (40.0)	24 (38.7)
Household includes an essential services person who continued to work outside the home	6 (50.0)	297 (51.4)
**COVID-19–like illness**		
COVID-19–like illness since January 1, 2020	8 (66.7)	257 (44.9)
**Symptoms among those reporting COVID-19-like illness**		
Cough (new onset or worsening of chronic cough)	6 (75.0)	159 (61.9)
Mostly dry cough	4 (80.0)	69 (85.2)
Headache	6 (75.0)	151 (58.8)
Altered sense of smell/taste	5 (62.5)	25 (9.7)
Sore throat	5 (62.5)	152 (59.1)
Fever (documented or subjective)	4 (50.0)	139 (54.5)
Runny nose (rhinorrhea)	4 (50.0)	134 (52.4)
Chills	3 (37.5)	10 (42.8)
Diarrhea	3 (37.5)	54 (21.0)
Exhaustion	3 (37.5)	118 (45.9)
Muscle aches (myalgia)	3 (37.5)	119 (46.3)
Shortness of breath (dyspnea)	3 (37.5)	80 (31.1)
Nausea or vomiting	2 (25.0)	34 (13.2)
Persistent pain or pressure in the chest	1 (12.5)	40 (15.6)
Diagnosed with COVID-19^e^	3 (37.5)	0

a *P* = .0448, χ^2^.

b *P* < .0001, χ^2^.

c Was within 2 m of a known/suspected COVID-19 patient for ≥15 min or lived with a known/suspected COVID-19 patient.

d *P* = .0072, χ^2^.

e *P* < .0001, χ^2^.

There were no statistically significant differences by serology status among employees reporting travel outside of the region or social distancing practices. Seropositive employees were more likely than seronegative employees to report having a known or suspected COVID-19 case in their household (41.7% vs 10.9%, *P* = .0072), and they were just as likely as seronegative employees to report living with an essential services worker who continued to work outside the home (50.0% vs 51.4%; *P* = .53). Although more seropositive employees had contact outside of work with a known or suspected COVID-19 contact, this difference was not statistically significant (41.7% vs 31.6%; *P* = .13).

Two-thirds of the seropositive employees (66.7%) reported a COVID-19–like illness since January 1, 2020, compared with 44.9% of seronegative employees, however this difference was not statistically significant (*P* = .13). Of the seropositive employees, 4 (33.3%) were asymptomatic. Among the 258 seronegative employees reporting COVID-19–like illness, 56 stated that their symptoms ended in January or February. Seropositive and seronegative employees showed different profiles in symptoms; seropositive employees were more likely to report sore throat, dry cough, and headache.

## Discussion

Our findings among employees in a rural healthcare network show that direct patient care was not associated with increased likelihood of COVID-19 infection and that seropositivity was more likely associated with nonwork exposures. Similar findings have been reported in urban, densely populated settings and larger medical centers.^3-7^ Although travel, social-distancing practices, and having an essential-services worker in the household did not differ by antibody status in our study, being exposed to a COVID-19 contact outside of work or in the same household was positively correlated with antibody status.

This study is potentially limited by the timing between COVID-19 exposure and the antibody test. Employees tested >2–3 months following COVID-19 infection may no longer have detectable levels of IgG antibodies, thereby underestimating the prevalence of previous employee infection.^8-10^ Other limitations include sampling and recall bias. Employee antibody testing was not done entirely at random; therefore, the estimate of seropositivity reported may not be representative of all employees. Also, seropositive employees may have been more likely to accurately recall potential exposures to COVID-19. Finally, due to the low prevalence of seropositivity, statistical comparisons between seropositive and seronegative employees had limited statistical power.

During government-mandated shelter-in-place orders, SARS-CoV-2 IgG seroprevalence among employees in a rural healthcare network was lower than for the community at large. In this rural region, healthcare workers were more likely to be exposed to COVID-19 outside of the workplace than on the job. Thus, it is important that healthcare workers maintain high vigilance regarding potential nonwork exposures as well as healthcare-related patient-care exposures.
